# Minocycline as a re-purposed anti-*Wolbachia* macrofilaricide: superiority compared with doxycycline regimens in a murine infection model of human lymphatic filariasis

**DOI:** 10.1038/srep23458

**Published:** 2016-03-21

**Authors:** Raman Sharma, Ghaith Al Jayoussi, Hayley E. Tyrer, Joanne Gamble, Laura Hayward, Ana F. Guimaraes, Jill Davies, David Waterhouse, Darren A. N. Cook, Laura J. Myhill, Rachel H. Clare, Andrew Cassidy, Andrew Steven, Kelly L. Johnston, Louise Ford, Joseph D. Turner, Stephen A. Ward, Mark J. Taylor

**Affiliations:** 1 Department of Parasitology, Liverpool School of Tropical Medicine, Pembroke Place, Liverpool L3 5QA, UK

## Abstract

Lymphatic filariasis and onchocerciasis are parasitic helminth diseases, which cause severe morbidities such as elephantiasis, skin disease and blindness, presenting a major public health burden in endemic communities. The anti-*Wolbachia* consortium (A·WOL: http://www.a-wol.com/) has identified a number of registered antibiotics that target the endosymbiotic bacterium, *Wolbachia,* delivering macrofilaricidal activity. Here we use pharmacokinetics/pharmacodynamics (PK/PD) analysis to rationally develop an anti-*Wolbachia* chemotherapy by linking drug exposure to pharmacological effect. We compare the pharmacokinetics and anti-*Wolbachia* efficacy in a murine *Brugia malayi* model of minocycline versus doxycycline. Doxycycline exhibits superior PK in comparison to minocycline resulting in a 3-fold greater exposure in SCID mice. Monte-Carlo simulations confirmed that a bi-daily 25–40 mg/Kg regimen is bioequivalent to a clinically effective 100–200 mg/day dose for these tetracyclines. Pharmacodynamic studies showed that minocycline depletes *Wolbachia* more effectively than doxycycline (99.51% vs. 90.35%) after 28 day 25 mg/Kg *bid* regimens with a more potent block in microfilarial production. PK/PD analysis predicts that minocycline would be expected to be 1.7 fold more effective than doxycycline in man despite lower exposure in our infection models. Our findings warrant onward clinical investigations to examine the clinical efficacy of minocycline treatment regimens against lymphatic filariasis and onchocerciasis.

Lymphatic filariasis (LF) and onchocerciasis are parasitic helminth diseases affecting an estimated 158 million people globally, causing a substantial socioeconomic and health burden in endemic communities^1^,^2^,^3^. Lymphatic filariasis, caused by the filarial nematodes *Wuchereria bancrofti, Brugia malayi* and *B. timori* affects an estimated 120 million people. Morbidity associated with LF includes lymphoedema, elephantiasis and hydrocele^1^,^3^. Onchocerciasis is caused by *Onchocerca volvulus* and affects approximately 38 million people. Symptomatically, onchocerciasis presents as skin disease and can cause loss of sight in advanced untreated cases due to the immune response to migration and death of microfilariae in the eye^2^,^3^.

Current filariasis treatment and control programs rely on the use of the microfilaricides diethylcarbamazine, ivermectin, and albendazole. In the context of mass drug administration (MDA), a combination of ivermectin and albendazole is used to control LF in Africa whereas a diethylcarbamazine/albendazole combination treatment is mainly used in the rest of the world^3^. In the case of onchocerciasis, ivermectin is administered as a monotherapy during MDA in all endemic areas. Targeting the microfilariae has proved effective in many cases, however, the length of treatment required (5 years for LF and up to 17 years or more for onchocerciasis) combined with long life span of an adult worm means that transmission will not be interrupted in some cases, especially where MDA is disrupted^3^,^4^. Treatment in geographical regions in which *Loa loa* is co-endemic is further complicated as ivermectin can cause severe adverse reaction events (SAEs), which can result in encephalophathy, coma and death^5^. These SAEs are probably caused by the drug-induced paralysis and death of the *Loa loa* microfilariae in the brain^6^. There is also increasing evidence of ivermectin resistance emerging in some endemic regions^7^. Epidemiological modelling also suggests MDA based on a microfilaricide mode of action may not be effective for elimination in all scenarios^8^,^9^, therefore alternative treatment strategies that include the use of macrofilaricidal (curative) drugs require development to facilitate the elimination of filarial diseases^10^.

Lymphatic filariae and *O. volvulus* are host to the obligate endosymbiotic bacterium, *Wolbachia,* which is essential for their development, growth and survival^10^. *Wolbachia* also contribute to filarial disease pathology^11^,^12^ and are associated with inflammatory adverse reactions to standard microfilaricidal treatments^13^,^14^. It has been shown that targeting *Wolbachia* with antibiotic drugs can induce growth retardation, embryotoxicity and death of adult filarial nematodes in preclinical models^15^,^16^ and in humans^17^. Doxycycline is the current gold standard anti-*Wolbachia* treatment with a growing body of clinical evidence that it has macrofilaricidal activity against LF^18^,^19^ and onchocerciasis^20^,^21^,^22^. Tetracyclines including doxycycline have been shown to suppress immune activity and therefore may offer the added benefit of alleviating symptomology^23^,^24^. This effect has been confirmed in clinical studies in which 200 mg/day doxycycline has been given for six weeks, showing reduced lymphangiogenic factors and filarial lymphedema^25^,^26^. Additionally, doxycycline is safe to use in *Loa loa* co-endemic areas as these parasites do not harbour *Wolbachia*^22^. The use of doxycycline in mass drug administration is however hampered by the duration of an optimally effective treatment (for onchocerciasis, recently defined as a minimum of 4 weeks, 100 mg, in a meta-analytical modelling framework) which could pose a hurdle to treatment adherence^27^.

The anti-*Wolbachia* consortium (A·WOL) program has identified a number of registered antibiotics that were effective against *Wolbachia* in *in vitro* and *in vivo* model systems^28^. The tetracycline, minocycline, caused a 1.6-fold log drop in *Wolbachia* in an *in vitro* C6/36 (*w*AlbB) cell based screen when administered at a concentration of 10 μM. A regimen of 25 mg/Kg/day of minocycline administered for 10 days elicited a log drop of 3.78 in *Wolbachia ftsZ* DNA in the *Litomosoides sigmodontis* murine filariasis model^28^. The data suggest superiority of minocycline over doxycycline. In this current work, we have investigated and compared the anti-*Wolbachia* efficacy of orally administered minocycline and doxycycline against the human filarial parasite *B. malayi* in a SCID mouse model^29^. Using quantitative assessment of *Wolbachia* load and PK/PD analysis we show that for a dosage that is equivalent to a previous clinically effective 100–200 mg/day doxycycline dose, minocycline is significantly more efficacious. The origin of these efficacy differences has been dissected through analysis of the relative contributions of drug pharmacokinetics and pharmacodynamics. Treatment duration and dosage have been investigated to identify optimal treatment regimens.

## Results

### *In vitro* anti-*Wolbachia* potency of minocycline *vs* doxycycline

The anti-*Wolbachia* potency of minocycline and doxycycline in *in vitro Wolbachia* infected *A. albopictus* cells C6/36 and *in vitro* male *Brugia malayi* worms harvested from untreated infected mice, is shown in Table 1. The data indicate that *in vitro* activities overlap for doxycycline and minocycline suggesting that both intrinsic antibiotic potency (*in vitro* data) and penetration of the filarial cuticle (*in vitro* worm data) are within the same range for both doxycycline and minocycline.

### Pharmacokinetics of doxycycline and minocycline in BALB/c SCID mice

**Figure 1:**
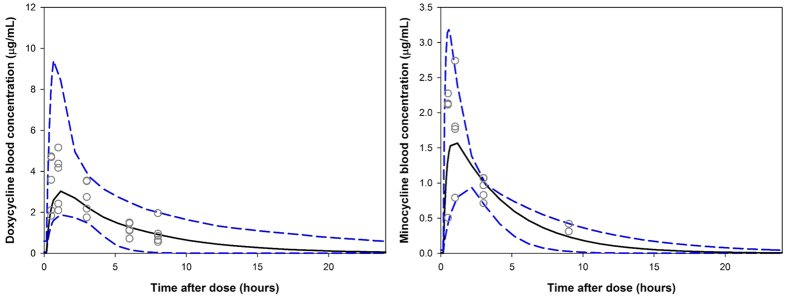
Pharmacokinetic profiles for doxycycline and minocycline at day 7. Solid black line represents the median for a population of 1000 simulated individuals and the blue dashed lines depict the 5^th^ and 95^th^ percentile values for the population. Observed discreet concentrations are shown as grey circles.

The pharmacokinetics of doxycycline and minocycline were determined for a 7 day 25 mg/Kg *qd* chronic dosing regimen (equivalent to the treatment regimens used in the murine infection studies). Table 2 shows the fitted pharmacokinetic profiles at steady state (day 7) for doxycycline and minocycline. Pharmacokinetic parameters for doxycycline day 1 were found to be not statistically different to those determined at steady state (Mann-Whitney test, n = 5, P-value > 0.05), this is consistent with there being no reports of time-dependent kinetics for tetracyclines in the literature. Figure 1 shows the predicted population PK profiles for doxycycline and minocycline. Minocycline was found to have a larger apparent clearance (CL/F: 3.2 vs. 1.2 L/h/Kg) and higher apparent volume of distribution than doxycycline (V/F: 13.2 vs. 6.9 L/hr). The elimination half-lives determined in BALB/c SCID mice were found to be comparable with those reported in the literature for doxycycline and minocycline in mice^30^,^41^. From Table 2 it can be seen that these differences lead to minocycline having approximately 3-fold less systemic exposure than doxycycline.

### Calculating murine dosage regimens bioequivalent to clinically relevant exposures

**Figure 2:**
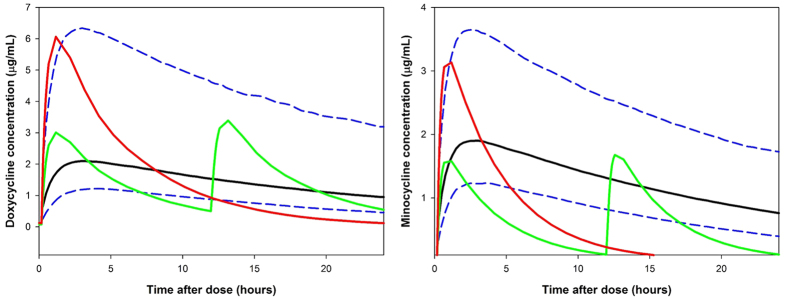
Concentration-time profiles for human and murine pharmacokinetic studies. Median human pharmacokinetic profile (200 mg dose) is shown as a solid black line and the blue dashed lines depict the 5^th^ and 95^th^ percentile values for a population simulated population of 1000 individuals. Median 25 mg/Kg *bid* and 50 mg/Kg murine pharmacokinetic profiles are show in green and red solid lines, respectively.

Monte Carlo simulations were used to calculate murine dosage regimens that give bioequivalent exposures to standard clinical 100–200 mg/day doses of second-generation tetracyclines. The distributions of human exposures of doxycycline and minocycline were based on pharmacokinetic parameters from the literature^31^,^32^. Figure 2 shows the simulated human pharmacokinetic profiles of 200 mg doses of doxycycline and minocycline overlaid with their respective bioequivalent murine pharmacokinetic profiles given as either once-daily or twice daily regimens. From simulation it has been determined that a 25 mg/Kg *bid* dosage regimen closely emulates overall daily exposure of a 100–200 mg clinical dose for both tetracyclines. For doxycycline oral doses of 100 mg and 200 mg in humans give mean exposures of 28.4 and 56.9 μg.hr/mL, whereas in mice 25 mg/Kg doses once or twice daily give mean exposures of 22.8 and 40.6 μg.hr/mL, respectively. For minocycline, 100 mg and 200 mg doses in the clinical yield exposures of 17.7 and 35.4 μg.hr/mL, whereas in mice 25 mg/Kg doses administered once or twice daily give exposures of 7.8 and 14.9 μg.hr/mL, respectively. Multiple daily doses are more preferable to higher dose once daily as this results in peak plasma concentrations that are more consistent with those most often achieved clinically with a standard doses of tetracycline and the length of exposure is more reflective of the clinical plasma concentration-time profile.

### Comparative *in vivo* efficacy of clinical bioequivalent doxycycline and minocycline regimens against adult stage *Brugia malayi Wolbachia*

**Figure 3:**
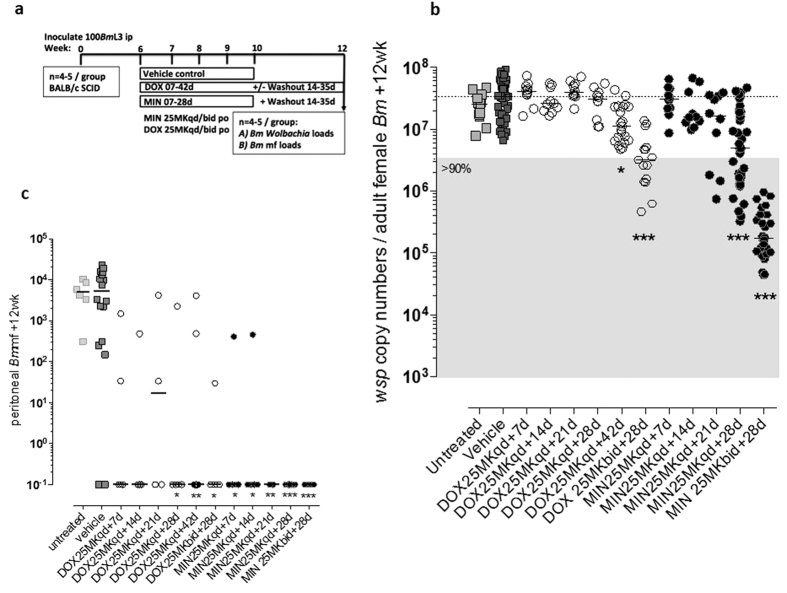
(**a**): Schematic of *Brugia malayi (Bm)* SCID mouse PD model with oral dosing regimens (MK: mg/Kg). (**b**): Effect on *Wolbachia* depletion following indicated human bioequivalent dosing of doxycycline (DOX) or minocycline (MIN). Data plotted is *Wolbachia surface protein (wsp)* single gene copy number per adult female worm, horizontal bars are median levels. Shaded area indicates levels above 90% depletion compared with median vehicle control level (dashed line). Data is derived from between 10 and 40 adult female worms pooled from groups of between 4–12 mice per drug treatment. Significant differences between the 13 treatment groups were identified by Kruskal-Wallis, two-tailed 1-way ANOVA, alpha = 0.05, *P* < 0.0001 with Dunn’s multiple tests post-hoc comparing drug groups vs vehicle control, indicated *P < 0.05, **P < 0.01 and ***P < 0.001. (**c**): Effect on microfilariae (mf) production following human bioequivalent dosing of doxycycline or minocycline. Data is total mf enumerated from the infection site in groups of 4 to 12 mice per drug group. Horizontal bars are median levels. Significant differences between the 13 treatment groups were identified by Kruskal-Wallis, two-tailed 1-way ANOVA, alpha = 0.05, *P* < 0.0001 with Dunn’s multiple tests post-hoc comparing drug groups vs vehicle control, indicated *P < 0.05, **P < 0.01 and ***P < 0.001.

To directly compare efficacy of minocycline against doxycycline, dosages determined bioequivalent to typical human exposures; 25 mg/Kg either once or twice daily, were orally administered to *B. malayi-*infected SCID mice (Fig. 3a). Dose durations were extended up to 42 days, to match proven macrofilaricidal 4–6 week doxycycline clinical dosage regimens (defined as depletion of >90% *Wolbachia* from nematode tissues)^14^,^33^.

Table 3 details the adult parasitological outcomes post-doxycycline or minocycline treatment. Approximately 90% (19/22) of vehicle control treated SCID mice harboured fecund, motile *B. malayi* at +12 weeks (median 6 worms/animal, range 0–19, sex ratio 59.2% female). As predicted for slow-acting macrofilaricides targeting *Wolbachia,* neither doxycycline nor minocycline, at any regimen tested, exerted a negative effect on adult filarial burdens at +12 weeks. Sex ratios were also not significantly distorted by drug treatment.

Median *Wolbachia* loads per female *B. malayi* were estimated at 2.57 × 10^7^ (range 7.74 × 10^6^–4.76 × 10^7^) derived from untreated SCID mouse infections. Filarial *Wolbachia* loads from vehicle control treated mice levels were also similar (median 3.35 × 10^7^, range 6.51 × 10^6^–9.33 × 10^7^). Doxycycline reduced *Wolbachia* in both a dose and dose-duration dependent-manner (Fig. 3b). In line with clinical data from doxycycline-treated LF patients, and confirming bio-equivalent dose calibrations, doxycycline mediated significant (>90% threshold) depletion of *Wolbachia* in 28 days, dependent on dose (median reduction 90.35%, 25 mg/Kg *bid* x 28d). In comparison, minocycline mediated improved efficacy with reduced dosages and/or dose-durations. A 25 mg/Kg *qd* minocycline regimen yielded significant, yet sub-optimal, reductions in *Wolbachia* following 28 day dosing, which was an improvement compared with matching doxycycline dose delivered for 42 days (median reduction 85.08%, minocycline x 28d *vs* 66.30%, doxycycline x 42d, *P = *0.0307, Mann-Whitney test). Increasing the dose of minocycline to 25 mg/Kg *bid* mediated a significant, above clinical threshold effect in depletion of >90% of *Wolbachia* following 28 day dosing, an improvement on matching doxycycline dose (median reduction 99.51%, minocycline *vs* 90.35%, doxycycline*, P* *<* 0.0001, Mann-Whitney test).

Depleting *Wolbachia* from filarial nematodes blocks embryogenesis^34^ and results in a waning of circulating LF mf in the blood or *O. volvulus* mf in the skin, which can be detected 2–4 months after start of effective doxycycline treatment^14^,^18^,^22^. Therefore, comparative effects of doxycycline and minocycline in blockading *B. malayi* embryogenesis were assessed *in vivo* by measuring accumulations of released mf at the peritoneal infection site (Fig. 3c). At 12 weeks post-infection, 89% of all vehicle treated mice contained mf (median 5396, range 0–23008). Doxycycline mediated a significant reduction in peritoneal mf loads following 28 or 42 days 25 mg/Kg *qd* doses and *bid* dosing of 25 mg/Kg for 28 days (Fig. 3c). However, at all doses and durations tested, at the evaluation endpoint blockade of embryogenesis was incomplete with between 20–50% of treated animals harbouring motile mf (Fig. 3c). In comparison, minocycline mediated significant reduction in peritoneal mf loads at all durations and doses tested, as little as 7 days at 25 mg/Kg *qd,* and rendered a complete blockade of embryogenesis after 3–4 weeks of exposure to 25 mg/Kg *qd* or *bid* regimens.

### Immediate anti-*Wolbachia* pharmacological effect of escalating minocycline dose regimens

**Figure 4:**
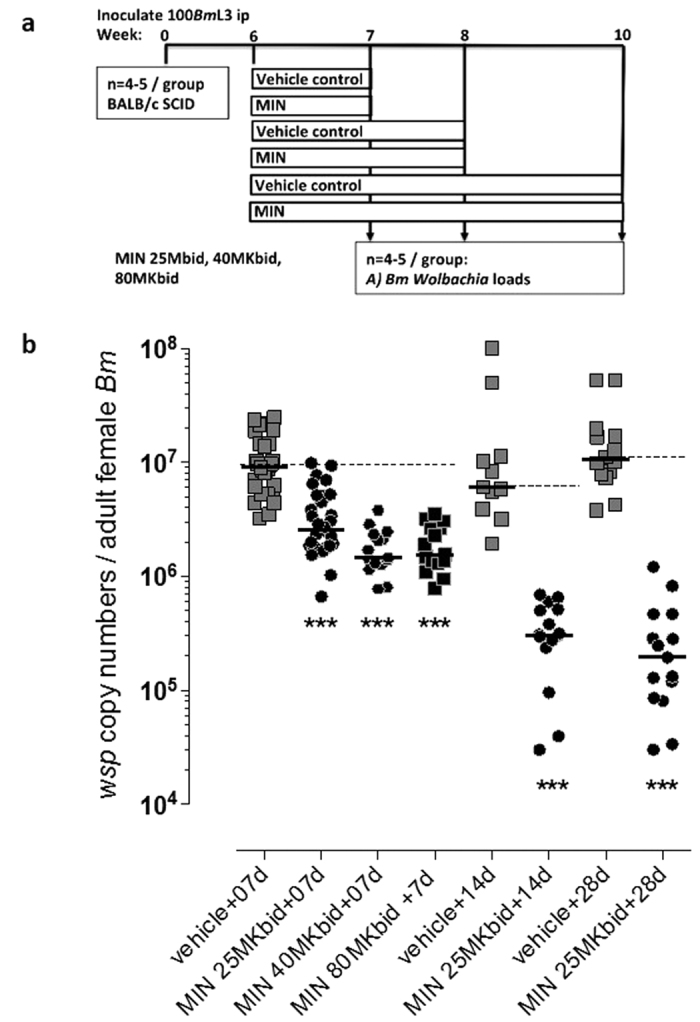
(**a**): Schematic of *Brugia malayi (Bm)* SCID mouse PD model with oral dosing regimens (MK: mg/Kg, ip: intraperitoneal route). (**b**): Effect on *Wolbachia* depletion following indicated minocycline (MIN). Data plotted is *Wolbachia surface protein (wsp)* single gene copy number per adult female worm, horizontal bars are median levels. Median vehicle control level is indicated by dashed line. Data is from 10–15 adult female worms pooled from between 5–10 mice per drug treatment. Significant differences between 7 day MIN dose escalation groups and vehicle were compared by Kruskal-Wallis, two-tailed 1-way ANOVA, alpha = 0.05, *P* < 0.0001 with Dunn’s multiple tests post-hoc comparing all groups indicated *P < 0.05, **P < 0.01 and ***P < 0.001. Significant differences between MIN 25MKbid for 14 or 28d vs corresponding length of vehicle were compared by Mann-Whitney tests, two-tailed, alpha = 0.05, *P* < 0.0001 indicated***.

To assess the immediate pharmacological effect of minocycline dose regimens (i.e. without the impact of a washout period, post antibiotic effect) adult *B. malayi* parasitized mice were dosed with oral minocycline at 25, 40 or 80 mg/Kg *bid* for 7 days or 25 mg/Kg *bid* for 14 or 28 days (Fig. 4a). Adult worms were recovered immediately following the last dose and *Wolbachia* loads in female adult nematode tissue was enumerated (Fig. 4b). Seven day dosing of 25, 40 or 80 mg/Kg *bid* lead to sub-threshold, yet significant, depletions of *Wolbachia* (71.65–84.08% median depletions, 25–80 mg/Kg *bid,* Kruskal-Wallis 1-way ANOVA *P* *<* 0.0001). There were no significant changes in the level of *Wolbachia* depletion when comparing dose escalations between 25–80 mg/Kg *bid* over a 7-day course by 1-way ANOVA. When comparing extended dose durations, both 2 week and 4 week exposures led to >90% *Wolbachia* depletions (94.93% and 98.17%, respectively).

### Pharmacokinetic-Pharmacokinetic modelling

PKPD modelling incorporating an effect compartment was employed to assess the rate of *Wolbachia* reduction. The anti-*Wolbachia* potencies at the effect site were set equal to potencies determined in previously validated assay using *Wolbachia* infected cells and transfer coefficients to the effect site were fitted using non-parametric non-linear mixed effects modelling. Table 4 shows *in vitro* potencies and fitted transfer coefficients for both tetracyclines. The combination of minocycline’s increased potency and faster transfer to the effect compartment leads to 1.7-fold greater anti-*Wolbachia* effect in comparison to doxycycline with the transfer rate into the effect site being 24-fold higher for minocycline.

## Discussions

The A∙WOL consortium aims to reduce the treatment time of anti-*Wolbachia* macrofilaricidal chemotherapies to improve test-and-treat drug regimen delivery, adherence and to reduce costs of treatment. Minocycline has been shown to have a similar anti-*Wolbachia* effect to doxycycline *in vitro* with improved *in vivo* efficacy, making it an ideal candidate for further PKPD investigation^28^.

Traditionally pre-clinical and clinical studies of anti-filarial chemotherapies have linked drug dosage to pharmacological effect^35^,^36^,^37^. Linking dosage to pharmacological effect when comparing chemotherapies is inherently flawed as the pharmacokinetics (absorption, distribution, metabolism and excretion) of different chemotherapies within a target population can vary radically and therefore the systemic exposure of the drug at the effect site can be significantly different for the same dose of two different drugs. This is an even greater problem in preclinical models where species and strain differences in PK can be radically different. The resultant pharmacological effect can therefore deviate for drugs with similar *in vitro* potencies. The use of rational drug development employing PKPD modelling or model-based drug development (MBDD) is a paradigm that has been used extremely effectively with preclinical and clinical development in other disease areas^38^,^39^,^40^. We propose, supported by the data presented here, that the NTD community, particularly those involved in the development of new chemotherapies, need to embrace these strategies if we are ever to optimise global treatment and elimination programmes to meet control and elimination targets.

In this work, for the first time, we use PKPD modelling to rationally develop an anti-filarial chemotherapy by linking drug exposure to pharmacological effect. Specifically, we use PKPD analysis to evaluate the relative efficacy of minocycline to the gold standard anti-*Wolbachia* macrofilaricide doxycycline in our *Brugia malayi* murine infection model of lymphatic filariasis. There is an inherent disconnect between the human pharmacokinetics and murine pharmacokinetics of tetracyclines with murine body weight normalised clearances being far greater than is the case in their human counterparts^30^,^31^,^41^. To address this disconnect we determined the murine pharmacokinetics of these tetracyclines, minocycline was found to have inferior pharmacokinetics to doxycycline, giving approximately 3-fold less exposure in comparison to doxycycline in our murine models. Monte-Carlo simulations of murine and human exposures were used to establish that a bi-daily 25 mg/Kg murine dose gave an approximately bioequivalent exposure to the currently recommended 100–200 mg daily clinical dose of the tetracycline. At bioequivalent exposures minocycline was found to deplete *Wolbachia* levels by >99% after a 28 day treatment regimen in the *Brugia malayi* murine model. This *Wolbachia* reduction is far greater than the 1 log-unit drop associated with irreversible sterilisation filarial tissues in clinical studies, the target clinical PD endpoint^14^,^33^. In contrast doxycycline at a 25 mg/Kg *bid* bioequivalent dosing regimen achieved a 90.35% reduction within the same 28 day time frame.

**Figure 5:**
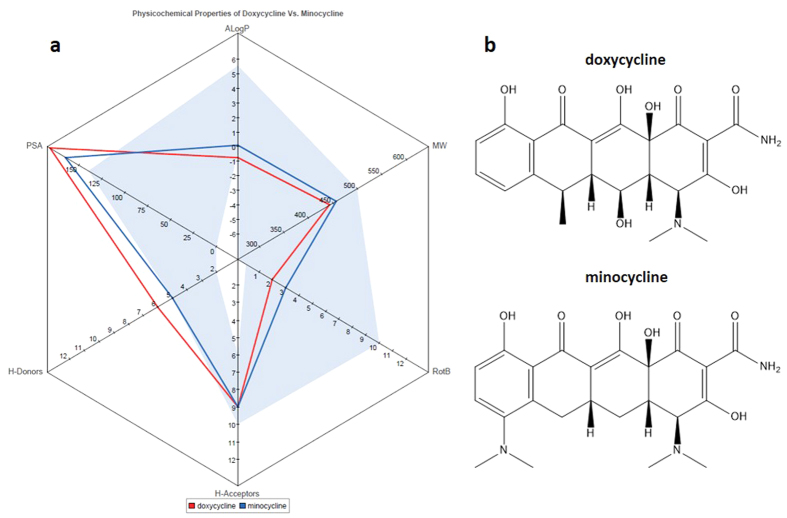
(**a**). Physicochemical properties of doxycycline and minocycline shaded regions represents those synonymous with favourable permeation. (**b**). Chemical structures of doxycycline and minocycline.

PKPD modelling was used to quantify that minocycline is 1.7-fold more effective than doxycycline when administered at the 25 mg/Kg *bid* bioequivalent dosage regimen, despite a 3-fold lower exposure than doxycycline and having a similar potency at the effect site as modelled by *in vitro* potencies. A PKPD model incorporating an effect compartment was employed; this enabled us to further dissect the origins of the superior efficacy of minocycline given its inferior systemic exposure and similar potency to doxycycline. These properties don’t account for the increase in efficacy indicating there are other factors influencing its *in vivo* efficacy. The transfer rates to and from the effect site were determined from the PKPD analysis, the transfer rate constant to the effect site was predicted to be 24-fold higher for minocycline in comparison to doxycycline whereas the efflux from the effect site was predicted to only marginal faster for minocycline. The difference in transfer rates in to the effect site could be due to increase bioavailability at the effect site from the systemic exposure or the transfer coefficient could be a lumped parameter masking the production of an active metabolite of minocycline that has a far greater potency than doxycycline. We have sought to explain the difference in transfer rate to the effect site through analysis of the physicochemical properties of doxycycline and minocycline focusing on physicochemical properties synonymous with permeability^42^,^43^, namely calculated octanol-water partition coefficient (ALogP), number of hydrogen bond acceptors/donors (H-Acceptor/H-Donor), molecular mass (MW), number of rotatable bonds (RotB) and molecular polar surface area (PSA). Figure 5 shows a radar plot of the physicochemical properties of doxycycline and minocycline, it can be seen that as expected the physicochemical properties of doxycycline and minocycline are similar, however there are notable differences. The optimal region for physicochemical properties with respect to permeability and oral bioavailability as defined by previous analyses of experimental *in vitro* and *in vivo* data^42^,^43^,^44^ are shown as shaded areas in Fig. 5. The physicochemical parameters of minocycline confer with more optimal regions of physicochemical space than doxycycline, in particular calculated LogP and number of hydrogen bond donors were sub-optimal for doxycycline in comparison to minocycline, furthermore minocycline’s molecular polar surface area (PSA) is more in-line with the recommend 140 Å^2^ cut-off limit for permeable compounds. These subtle differences may lead to a change in the transfer rates into the effect site.

The overall conclusion of this study is that minocycline is predicted to be superior to doxycycline as an anti-*Wolbachia* dependent macrofilaricide. The data suggest that not only can minocycline be used as an alternative to doxycycline in test and treat strategies it may offer some advantages through either improved *Wolbachia* kill rates or lower dosage requirements, which may impact on tolerability or safety profiles. Although the incidence of AEs with either drug is very low there is a slightly higher incidence of AE with minocycline^45^. The shorter treatment period may offset this slight increase in risk of AE with minocycline. It will now be important to undertake clinical evaluations of minocycline, supported through full PK analysis, to confirm these benefits. Recently, a clinical trial has shown a single dose triple combination of diethylcarbamazine, albendazole, and ivermectin has shown microfilaremia suppression in Bancroftian Filariasis for up to a year or more. These results are clearly promising, with the potential to accelerate MDA endpoints in non-African settings, where co-endemicity with either *Loa loa* or *Onchocerca volvulus* do not present a risk of severe adverse events. Ultimately, a number of chemotherapeutic tools will be need to eliminate filariasis, including anti-*Wolbachia* agents for settings where standard anti-filarials are contraindicated or compromised by reduced efficacy^46^.

We used PKPD modelling to ensure that drug exposures for our dosing regimens in our *Brugia malayi* preclinical murine model are indicative of those of recommended clinical doses, given the *Wobachia* reduction achieved at bioequivalent doses of minocycline. We recommend that further clinical studies investigating the anti-*Wolbachia* effect of 2–4 weeks of 100 or 200 mg/day minocycline in patient population with lymphatic filariasis and onchocerciasis. Furthermore, treatment time maybe further truncated by combination with other classes of anti-*Wolbachia* registered drugs or in combination with current anti-filarial drugs, which are actively being pursued by A∙WOL.

## Methods

### Parasites

The parasite life cycle of *B. malayi* (*Bm*) filarial nematode (TRS strain)^47^ was maintained in mosquitoes and susceptible *Meriones unguiculatus* gerbils at LSTM. Infective *Bm* larvae (*Bm*L3) were generated using protocols as previously described^29^. Briefly, female adult *Aedes aegypti* mosquitoes were fed with microfilariae collected from infected gerbils by catheterisation, as described previously^48^. Microfilariae were mixed with human blood and fed to mosquitoes using an artificial membrane feeder (Hemotek^®^). *Bm*L3 stage larvae were propagated by rearing the blood-fed mosquitoes for 14 days. *Bm*L3 larvae were then harvested from infected mosquitoes by crushing. Purification of the inoculum was performed using previously optimised protocols^25^.

### Animals

Male BALB/c SCID mice were procured from Harlan Laboratories, UK. All animals were housed at an approved animal housing facility under specific pathogen-free (SPF) conditions. Approval was obtained for all animal experiments from the ethical committees of the University of Liverpool and LSTM. Experiments were conducted according to Home Office (UK) requirements.

### Parasite Infections

100 freshly collected, motile *Brugia malayi* L3 larvae were injected via the intra-peritoneal route into BALB/c SCID mice. Efficiencies of inoculations were confirmed by needle washout.

### Preparation and administration of Drugs/Compounds

Infected mice were administered 100 μL of either doxycycline or minocycline solution via oral gavage six weeks after infection. Doxycycline was administered once or twice daily at 25 mg/Kg for 7, 14, 21, 28 or 42 days. Minocycline was administered in dosage regimens mirroring those for doxycycline. Additionally, minocycline was administered twice daily at 25, 40 and 80 mg/Kg for 7 days. Both doxycycline and minocycline were dissolved in water and all reagents were purchased from Sigma unless otherwise stated.

### Parasitological Readouts

Mice were necropsied 12 weeks after infection unless otherwise stated. A total of 5 individual screens were run, each containing groups of between 4/5 SCID mice per treatment arm. Peritoneal washes using RPMI medium were employed to recover adults and microfilariae (mf). Adult worms were removed and stored overnight in fresh medium at 4 °C before being washed in cold PBS. Adult stages were subsequently counted and their gender determined. For subsequent qPCR analysis of *Wolbachia* loads, 10–15 female adults were utilised per treatment group, derived from each screen. Between 2–4 female worms were selected, randomly, from individual mice, to avoid bias due to intra-group dosing variation. Mf were pelleted from peritoneal washings by centrifugation (300 g, 5 minutes, 4 °C, low brake), re-suspended in 1–2 ml of medium and the total mf quantity contained within peritoneal lavage enumerated by microscopy.

### Quantification of *Wolbachia* bacteria numbers using qPCR assay

Extraction of DNA from individual female worms was performed using a DNeasy Blood and Tissue Kit (Qiagen) according to manufacturer’s instructions. Quantification of *Bm Wolbachia wsp* copy numbers was performed using qPCR as previously described^49^.

### Determination of *in vitro* potencies

The anti-*Wolbachia* potency of doxycycline and minocycline for use in the PKPD model was determined *in vitro*, utilising the routine A·WOL screening assay as described previously^50^. In brief the mosquito (*Aedes albopictus*) derived cell line (C6/36), stably infected with *Wolbachia pipientis* (*w*AlbB) (C6/36 (*w*AlbB)) was incubated with the relevant drugs in a concentration range in order to determine a dose response. The drugs were incubated for 7 days with 2,000 cells per well on a 384 well plate (CellCarrier-384 Ultra, PerkinElmer) in Leibovitz media (Life Technologies™) supplemented with 20% foetal bovine serum (FBS, Fisher Scientific), 2% tryptose phosphate broth (Sigma-Aldrich) and 1% non-essential amino acids (Sigma-Aldrich). The end-point read out utilised DNA staining of both the host cell nuclei and intracellular *Wolbachia* (SYTO^®^11) combined with a high content imaging system (Operetta^®^, PerkinElmer) and analysed using the associated Harmony^®^ software through a cytoplasm texture analysis.

### Pharmacokinetic studies in BALB/c SCID mice

Rich pharmacokinetics studies were performed to characterise the pharmacokinetic profiles of orally administered doxycycline and minocycline in uninfected BALB/c SCID mice (weight 24–28 g). 25 mg/Kg of doxycycline or minocycline was administered via oral gavage in the appropriate vehicles (PEG300/Propylene Glycol/H20 (55%,25%,20%)). Blood samples were collected via microsampling from the tail vein. For both drugs, serial blood samples were collected up to 8 hours after a single dose and on day 7 after once daily dosing.

Blood samples (20 μL) were collected via a pipette with a pre-heparinised tip and were immediately lysed with 40 μL of ice cold ddH_2_O and subsequently frozen at −80 ^o^C until time of analysis. Sparse samples were obtained from the pharmacodynamic studies in the *Brugia malayi* BALB/c SCID murine infection model to ensure that drug exposures were in line with those observed in the rich pharmacokinetic studies.

### Bioanalysis

Doxycycline and minocycline plasma drug concentrations were quantified using LCMS (liquid chromatography mass spectrometry) on a UHPLC (ultrahigh pressure liquid chromatography) system linked to a triple-quadruple TSQ Quantum Access mass spectrometer (Thermo Scientific, Hemel Hempstead, UK) with a heated-electrospray ionization (H-ESI) source. Chromatographic separation was performed using a gradient via a Hypersil C18 Gold column (100 × 2.1 mm, 1.9 μm particle size) reverse-phase column (Thermo Scientific, UK). All methods incorporated the use of appropriate internal standards and were validated using FDA guidelines which are internationally recognised^51^.

For both doxycycline and minocycline samples were prepared as follows: 20 μL of blood sample containing the drug was extracted with 120 μL acetonitrile/methanol (80%/20%) containing the internal standard (doxycycline and minocycline at a concentration of 1 μg/mL were employed as internal standards for each other). Samples were then filtered using 96 well filter plates (MultiScreen Solvinert, 0.45 μm hydrophobic PTFE filter plates, Millipore Ltd, Ireland) and the filtrates were transferred to clean glass autosampler vials for analysis. Samples were assayed alongside a whole blood calibration curve (range 0 - 50 μg/mL), with quality control samples at low 0.5 μg/mL, medium 15 μg/mL, and high 40 μg/mL concentrations for both doxycycline and minocycline. All standard curves were described using an equal weighted linear regression equation using data acquisition software LC Quan (Version 2.5.6.Thermo Scientific, Hemel Hempstead, UK). The correlation coefficient (r^2^) for both DOX and MN calibration curves exceeded 0.99. Lower limits of quantification for doxycycline and minocycline were 250 ng/mL and 300 ng/mL, respectively, with a signal-to-noise ratio of 5:1 and a coefficient of variation (CV) of less than 13% for both tetracyclines.

### Pharmacokinetics-Pharmacodynamic Modelling

All pharmacokinetic and pharmacodynamics modelling and simulation was performed using Pmetrics^52^ within R version 3.1.0^53^ A one-compartment model with an absorptive compartment for oral dosing was used for modelling the pharmacokinetics of doxycycline and minocycline as described by the differential equations 1a and 1b:


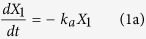



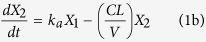


where X_1_ and X_2_ are the amounts of drug in the absorptive and central compartments, representing gut and systemic circulation, respectively. The pharmacokinetic parameters ka, CL, V represent the absorption rate constant, clearance and volume of distribution, respectively.

The link between drug exposure and pharmacological effect was quantified using PK/PD modelling. The pharmacodynamics response for the anti-*Wolbachia* effect exerted by doxycycline and minocycline was defined to be number of *Wolbachia* in each worm normalised to the relevant vehicle treated control and expressed as a percentage reduction. An effect compartment, X_3_, was used to account for the delay in observed effect as has been previously recommended^54^,^55^,^56^ and as defined by the differential equations 2a and 2b:


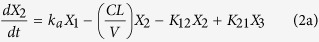



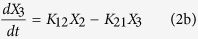


the parameters K_12_ and K_21_ in equations 2a and 2b represent transfer rate constants to and from the effect compartment. The pharmacodynamics response was modelled using an E_max_ model (equation 3) that depends on drug concentration in the effect compartment.


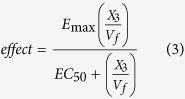


where EC_50_, E_max_, and V_f_ represent the maximum effect, the concentration to achieve 50% of the maximal effect and the effect site volume of distribution. The relative EC_50_ at the effect site was fixed to the *in vitro* EC_50_ determined in *Wolbachia* infected *A. albopictus* cells, E_max_ 99.999% and V_f_ is assumed to be proportional to the volume of distribution of the central compartment. The transfer rate constants, K_12_ and K_21_ to and from the effect compartment were fitted for each tetracycline.

Model fitting was performed via protocol used previously established protocols^57^,^58^ Briefly, linear regression (intercept close to 0, slope close to 1) was used to assess the goodness-of-fit of the observed/predicted values, the coefficient of determination of the linear regression and log-likelihood values. A statistically significant improvement in the log-likelihood value (P, 0.05) was required for a more complex model to be supported.

### Statistical tests

Continuous variables (*wsp* copy number/female *B. malayi* worm and total peritoneal mf/SCID mouse) were pooled per treatment group from independent screens. Statistical significance of differences between independent groups was assessed using two-tailed non-parametric statistical hypothesis. The Mann-Whitney U test was employed when comparing two groups whereas the Kruskal-Wallis test followed by Dunn’s multiple comparisons test, post-hoc, was used in the case of comparing more than two groups. Significance level was set to alpha = 0.05.

## Additional Information

**How to cite this article**: Sharma, R. *et al*. Minocycline as a re-purposed anti-*Wolbachia* macrofilaricide: superiority compared with doxycycline regimens in a murine infection model of human lymphatic filariasis. *Sci. Rep.* **6**, 23458; doi: 10.1038/srep23458 (2016).

## Figures and Tables

**Table 1:** *In vitro* anti-*Wolbachia* activities of doxycycline and minocycline in infected *A. albopictus* cells C6/36 and male *Brugia malayi* worms.

	Doxycycline	Minocycline
*In vitro* depletion (insect *Wolbachia*) EC_50_, (range, n)	15.3 nM (1–147, 396)	11.8 nM (8–34, 6)
*In vitro* depletion (male *B. malayi*) @5 μM Median% depletion cf vehicle, (range, n)	65% (14–80%, 10)	66.7% (43–71%, 10)

**Table 2:** Pharmacokinetic parameters for doxycycline and minocycline at steady state (day 7).

	Doxycycline	Minocycline
**CL/F (L/hr/Kg)**	1.2 (0.4)	3.2 (0.3)
**V/F (L/Kg)**	6.9 (3.9)	13.2 (4.5)
**Half-life**	4.4 (3.7)	3.0 (2.3)
**AUC (mg.hr/L)**_**0**–**24hr after dose**_	22.8	8.4

Standard deviations are show in parentheses.

**Table 3:** Parasitological output from comparative *in vivo* efficacy of clinically bioequivalent doxycycline and minocycline regimens against adult stage *Brugia malayi Wolbachia*.

Treatment	none	Vehicle (Water)	Doxycycline					Minocycline					
Dose	–	**–**	**25MKqd**	**25MKqd**	**25MKqd**	**25MKqd**	**25MKbid**	**25MKqd**	**25MKqd**	**25MKqd + 14d**	**25MKqd**	**25MKqd + 28d**	**25MKbid + 28d**
duration	–	**+28/42d**	**+7d**	**+14d**	**+21d**	**+28d**	**+28d**	**+42d**	**+7d**		**+21d**		
SCID n	7	22	5	4	4	5	5	9	5	5	5	13	5
**Adult *B. malayi***													
min	1	0	14	7	11	6	1	1	0	1	8	0	0
Median^	3	6	21**	9	15	9	6	6	19	10	12	6	6
max	5	19	22	11	19	13	10	21	23	21	20	15	10
total	22	122	96	36	60	49	28	69	76	55	70	81	49
													
**Female *B. malayi*(% total)**													
min	20.0	0	35.7	20.0	43.8	44.4	45	36.4	0	1	8	0	0
Median|	70.8	59.2	52.9	59.8	46.4	50.0	83	61.8	50	60.0	54.6	66.7	70.7
max	100	100	63.6	72.7	57.1	63.0	100	100	62	100	63.2	100	100
Female *B. malayi* n	13	68	51	19	29	25	17	40	38	30	38	53	32
*Wolbachia* sample n	13	40	10	10	10	10	10	21	10	10	10	35	21

^^^Kruskal Wallis 1way ANOVA *P* = 0.003, DOX 25MKqd +7d > Vehicle.

^**^P < 0.01, all other regimens *ns.*

^|^Kruskal Wallis 1way ANOVA *ns.*

**Table 4:** PKPD modelling parameters for doxycycline and minocycline.

	Effect site EC50 (nM)	K_12_/K_21_	Ratio of AUC_PD curve (0→28d)_
Doxycycline	15.32	4.36 × 10^−7^/8.06 × 10^−4^	1
Minocycline	11.75	1.08 × 10^−5^/1.49 × 10^−3^	1.7

Area under the PD curve for a 28 day 25 mg/Kg *bid* dosing regimen is also shown.
