# Corrigendum: Minocycline as a re-purposed anti-*Wolbachia* macrofilaricide: superiority compared with doxycycline regimens in a murine infection model of human lymphatic filariasis

**DOI:** 10.1038/srep46934

**Published:** 2018-01-08

**Authors:** Raman Sharma, Ghaith Aljayyoussi, Hayley E. Tyrer, Joanne Gamble, Laura Hayward, Ana F. Guimaraes, Jill Davies, David Waterhouse, Darren A. N. Cook, Laura J. Myhill, Rachel H. Clare, Andrew Cassidy, Andrew Steven, Kelly L. Johnston, Louise Ford, Joseph D. Turner, Stephen A. Ward, Mark J. Taylor

Scientific Reports 6: Article number: 2345810.1038/srep23458; published online: 03 21 2016; updated: 01 08 2018

The original version of this Article contained a typographical error in the spelling of the author Ghaith Aljayyoussi, which was incorrectly given as Ghaith Al Jayoussi. This has now been corrected in the PDF and HTML versions of the Article.

